# Primary Sequence and Three-Dimensional Structural Comparison between Malanin and Ricin, a Type II Ribosome-Inactivating Protein

**DOI:** 10.3390/toxins16100440

**Published:** 2024-10-13

**Authors:** Yan Yuan, Shuxiao Wu, Philip J. R. Day

**Affiliations:** 1 Key Laboratory of Chemistry in Ethnic Medicinal Resources, State Ethnic Affairs Commission & Ministry of Education, Yunnan Minzu University, Kunming 650500, China; 2 TELI College, Beijing Institute of Technology, Beijing 100081, China; shxw9876@126.com; 3 The Manchester Institute of Biotechnology, Faculty of Biology, Medicine & Health, University of Manchester, Manchester M13 9PL, UK; 4 The Medical Faculty, University of Cape Town, Rondebosch 7925, South Africa

**Keywords:** *Malania oleifera*, ribosome-inactivating protein, ricin, amino acid sequences, 3D structure

## Abstract

Malanin is a new type II ribosome-inactivating protein (RIP) purified from *Malania oleifera*, a rare, endangered tree is only found in the southwest of Guangxi Province and the southeast of Yunnan Province, China. The gene coding sequence of malanin was found from the cDNA library of *M. oleifera* seeds by employing the ten N-terminal amino acid sequences of malanin, DYPKLTFTTS for chain-A and DETXTDEEFN (X was commonly C) for chain-B. The results showed a 65% amino acid sequence homology between malanin and ricin by DNAMAN 9.0 software, the active sites of the two proteins were consistent, and the four disulfide bonds were in the same positions. The primary sequence and three-dimensional structures of malanin and ricin are likely to be very similar. Our studies suggest that the mechanism of action of malanin is expected to be analogous to ricin, indicating that it is a member of the type II ribosome-inactivating proteins. This result lays the foundation for further study of the anti-tumor activities of malanin, and for the application of malanin as a therapeutic agent against cancers.

## 1. Introduction

*Malania oleifera* Chun and Lee is a tree which grows to 10 to 20 meters in height, belonging to the monotypic genus *Malania* of the Olacaceae family [1,2]. This tree is rare and endangered [3,4,5] and grows in a restricted area within the Karst topography of southwest Guangxi and southeast Yunnan provinces, China [6,7].

The seeds of *M. oleifera* have a very high oil content (64.5%) and are used locally for making edible oils [8,9]. The seeds are distinctive for their high level of 15c-tetracosenoic acid (C24:1Δ15), a long-chain monounsaturated fatty acid, namely, a nervonic acid (C_24_H_46_O_2_, over 55.7–67% of total fatty acids) [10,11]. Nervonic acid is an important component in myelin biosynthesis in the central and peripheral nervous system. Myelin is generally localized to the sphingomyelin of animal cell membranes, where it has been proposed to enhance human brain function [12].

*M. oleifera* seeds also produce the protein malanin, a novel plant protein with a molecular weight of 61,875 Da and an isoelectric point of pH 5.5. Malanin was purified from *M. oleifera* seeds by homogenization, ammonium sulfate precipitation, and hydrophobic interaction chromatography. It is a glycoprotein with two chains, chain-A and chain-B, which are linked together through a disulfide bridge. Malanin has very strong anti-tumor activities and can not only inhibit protein synthesis in eukaryotic cells, but also induce an apoptotic response on human cervical cancer cells. However, the structure of malanin is unknown [13].

Ribosome-inactivating proteins (RIPs) are plant proteins that can inactivate ribosomes in eukaryotic cells and inhibit protein synthesis. They have biological activities such as anti-tumor, antiviral, immune regulation, and bone marrow purification [14,15]. According to their different primary structures, RIPs are divided into two types, single-chain and double-chain, named type I and type II. Type I RIPs are widely distributed and present in most organs of many plants, while type II RIPs are relatively rare [16].

The biggest difference between type I and type II RIPs is that type II RIPs have a binding chain-B, which can bind to D-galactose on the cell surface and assist in the entry of the A chain into the cell. The entire process is accomplished through endocytosis regulated by receptors. When type II RIPs enter the Golgi apparatus, the disulfide bond between the two chains is opened by protein disulfide oxidoreductase, and the independent A chain, which possesses extremely strong toxicity, can inhibit protein synthesis. The entire process is catalyzed with high efficiency by the enzyme [17,18]. Type I RIPs only have one catalytic chain A and no binding chain B, making them unable to bind to cells or enter the cell’s interior, resulting in poor inhibition of protein synthesis. In summary, type I RIPs exhibit extracellular toxicity, while type II RIPs exhibit lethal intracellular toxicity [19].

Malanin contains a catalytic A chain and a lectin-like B chain, and it is a member of type II RIPs [20]. Previous studies have found that the IC_50_ values of malanin in vitro Vero cells and MDCK cells were 2.79 × 10^−10^ mol/L and 3.92 × 10^−10^ mol/L, respectively, and showed dose and time dependence. The results of acute toxicity testing in vivo demonstrated that the LD_50_ values of malanin administered by gavage and intraperitoneal injection in mice were 43.11 ± 16.26 mg/kg and 26.22 ± 9.03 µg/kg, respectively. Both in vitro and in vivo results indicated that the toxicity of malanin is very strong, which limits its application in anti-tumor activities in vivo.

In this study, the gene coding sequence and amino acid sequence of malanin were found from the cDNA library of *M. oleifera* seeds [12] based on the 10 N-terminal amino acid sequences of malanin, which are DYPKLTFTTS in chain-A and DETXTDEEFN in chain-B. We were the first to report the peptide sequences of malanin, and have since studied the complete primary amino acid sequence of malanin and found it to be homologous to that of ricin. We also determined that abrin and cinnamonin share a close homology using the NCBI online Blastp (protein-protein BLAST) tool [21,22,23]. A crystal structure for ricin, which includes ricin A chain and ricin B chain, was previously determined from X-ray diffraction data [24,25,26]. The A chain inhibited protein synthesis, while the B chain directed and internalized the A chain into the cytoplasm by binding to the cell surface receptors carrying galactose. Based on the sequence homologies of these proteins, we fitted the primary sequence of malanin to the backbone structure for ricin and generated energy-minimized molecular models [27]. These models should prove particularly useful in studying the structure–function relationships of these proteins and for related RIPs in general.

## 2. Results and Discussion

### 2.1. Homologous Alignment of Malanin

**Figure 1:**
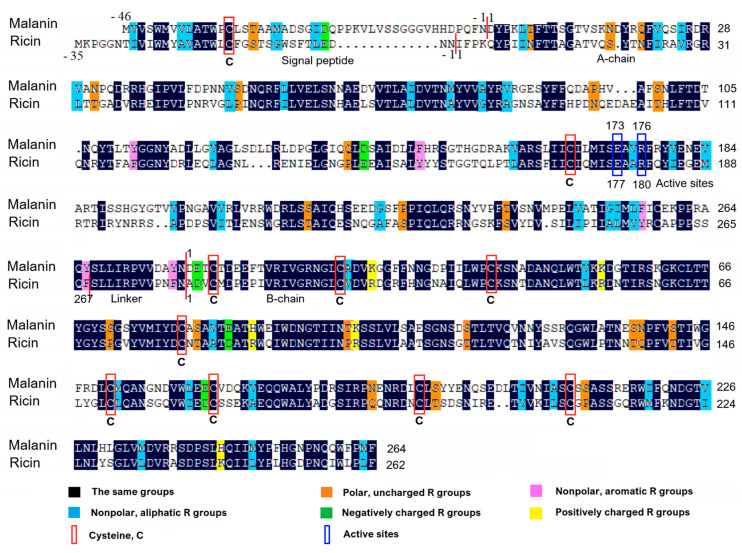
The homologous alignment between malanin and ricin was analyzed using DNAman 9.0 software. Among all the amino acid residues, the same was shown in black, those belonging to nonpolar and aliphatic R groups are shown in blue, those belonging to nonpolar and aromatic R groups are shown in purple, those belonging to polar and uncharged R groups are shown in orange, those belonging to positively charged R groups are shown in yellow, and those belonging to negatively charged R groups are shown in green. All cysteines are framed by red rectangles, and the active sites are framed by dark blue rectangles. Amino acids are numbered from the N-terminal residue of the mature A chain and B chain, and the preceding residues are indicated by negative numbers.

The BLASTP search analysis showed that malanin has a very high homology with type 2 RIP families. Among them, malanin shares the highest homology to ricin (Figure 1), which is 65% when similar activity amino acids are compared and 51% if only exact amino acid matches are included (Figure 1). The analogous amino acid residues between malanin and ricin were marked in black; nonpolar and aliphatic R groups in blue; nonpolar and aromatic R groups in purple; polar and uncharged R groups in orange; positively charged R groups in yellow; and, finally, negatively charged R groups were shown in green.

We previously reported that malanin is a glycoprotein with two chains, chain-A and chain-B, which are linked by one or more disulfide bonds. The amino acids are numbered from the N-terminal residue of the mature A chain and B chain, and the preceding residues are indicated by negative numbers. As shown in Figure 1, malanin has a total of 588 amino acid residues. Among them, the A chain has 278 residues, and the B chain has 264 residues. The signal peptide is believed to possess 46 amino acids.

### 2.2. The Phylogenetic Relationship between Malanin and Other Type II RIPs

Amino acid sequences of type 2 RIPs were aligned using ClustalX (1.83), and a phylogenetic tree was generated using the neighbor-joining method. The type 2 RIPs are listed in Table 1.

**Figure 2:**
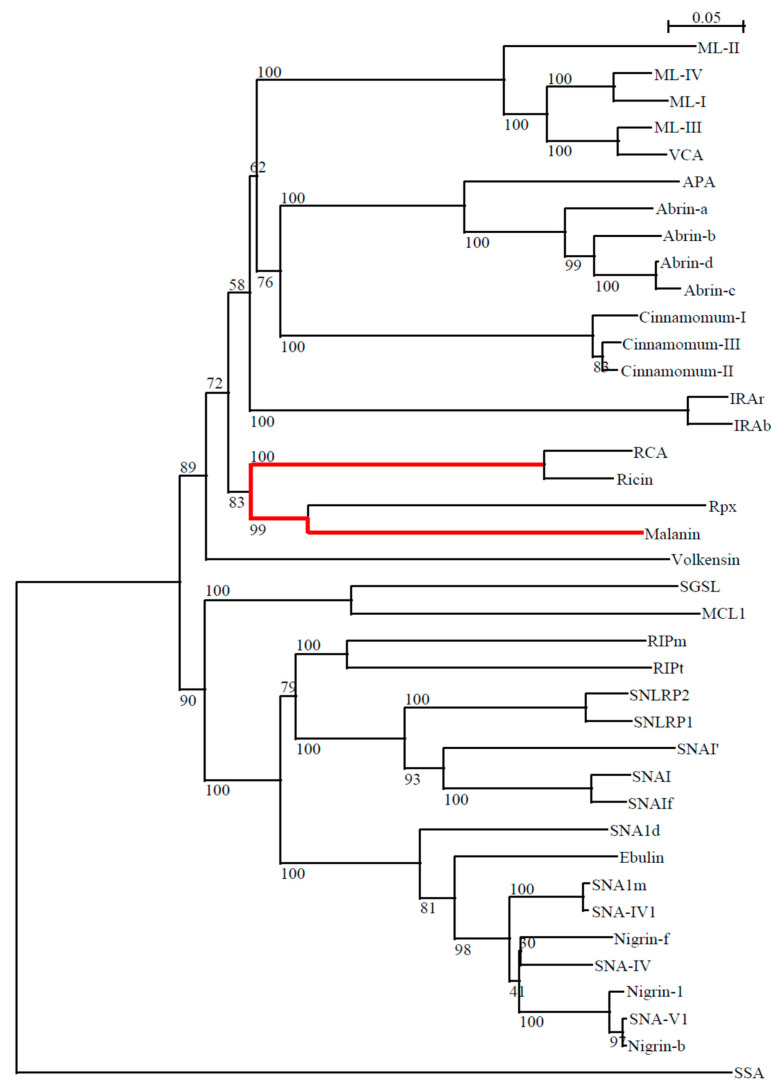
Dendrogram of the phylogenetic relationship of type 2 RIPs. Swiss-Prot/TrEMBL accession numbers: Malanin, Rpx Q2PA54, Ricin P02879, RCA P06750, Cinnamomin I Q94BW5, CinnamominII Q94BW4, CinnamominIII Q94BW3, Abrin-a P11140, Abrin-b Q06077, Abrin c P28590, Abrin-d Q06076, APA Q9M6E9, IRAb Q8W2E7, IRAr Q8W2E8, Ebulin l Q9AVR2, Nigrin b P33183, Nigrin l Q8GT32, Nigrin f O04367, SNA-IV O04366, SNA-IVl Q945S4, SNA-Vl Q945S2, SNAlm Q8GTA5, SNAld Q8GTA6, SNAIf O22415, SNAI Q41358, SNAI’P93543, SNLRP1 O04072, SNLRP2 O04071, SSA D25317 (GenBank), RIPt Q9M653, RIPm Q9M654, VCA Q8W243, ML I P81446, ML II Q6H266, ML III P82683, ML IV Q6ITZ3, MCL1 B7X8M2, SGSL U3KRF8, Volkensin Q70US9.

Figure 2 shows a phylogenetic tree of type 2 RIPs obtained by comparison of the sequences of some representative RIPs. The RIPs of the same species are also related. We selected significant RIPs of each known RIP-containing species to simplify the figure and also because the RIPs obtained from the same species, in general, show a higher homology among them than with RIPs of other species. Usually, RIPs of the same taxon have related amino acid sequences, thus indicating their presence in parental species and that they evolve in parallel to the differentiation of the corresponding plants. From Figure 2, we visualize that malanin has a very close relationship with ricin according to the phylogenetic tree (shown in red in Figure 2).

### 2.3. Three-Dimensional Structure of Malanin

**Figure 3:**
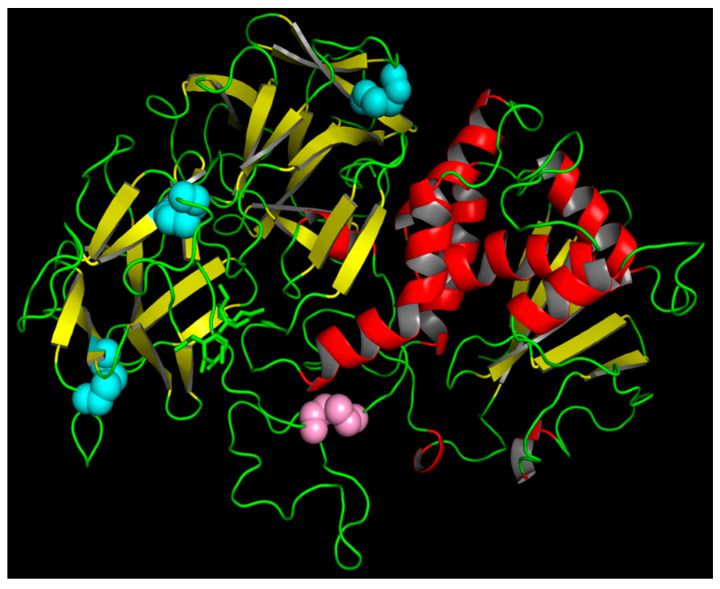
The three-dimensional structure of malanin. The structure of malanin created by Swiss-model and viewed by PyMOL 2.3. The α-helices, β-sheets, and loops are shown in red, yellow, and green, respectively. The blue spheres are disulfide bonds within the B chain, and the disulfide bond in pink links the malanin A chain to the B chain.

The three-dimensional structure of malanin was created by Swiss-model and viewed by PyMOL 2.3 (Figure 3). The α-helices, β-sheets, and loops are shown in red, yellow, and green, respectively. The blue spheres signify disulfide bonds within the B chain, and the disulfide bond in pink links the A chain to the B chain of malanin. As shown in Figure 3, there were four disulfide bonds in the three-dimensional structure of malanin. The pair of disulfide bonds in pink connects the A and B chains, and the other three pairs of disulfide bonds in blue form in the B chain. It is shown that malanin is a protein constructed from two peptide chains, chain-A and chain-B, which are crosslinked by a disulfide bond.

The Swiss-model template library (SMTL version 9 January 2020, PDB release 3 January 2020) was searched with BLAST (Camacho et al. [59]) and HHBlits (Remmert et al. [60]) for evolutionary-related structures matching the target sequence. PDB entry cinnamonin III (2vlc.1.A) was the best-scored template for both alternative search algorithms, with a GMQE (Global Model Quality Estimation) of 0.74, while the second and third highest scores were ricin and abrin, with GMQE scores of 0.68 and 0.64, respectively. Cinnamonin III, ricin, and abrin all belong to type II RIPs, which consist of two polypeptide chains, called A and B chains, which are linked together through a disulfide bridge. The A chain possesses rRNA N-glycosylase and polynucleotide adenosine glycosidase activities that irreversibly damage rRNA and other polynucleotide substrates inside the cells, thus causing cell death. The B chain has lectin properties, which allows type II RIPs to bind the galactoside residues on the cell membrane, facilitating entry into cells and resulting in high cytotoxicity.

**Figure 4:**
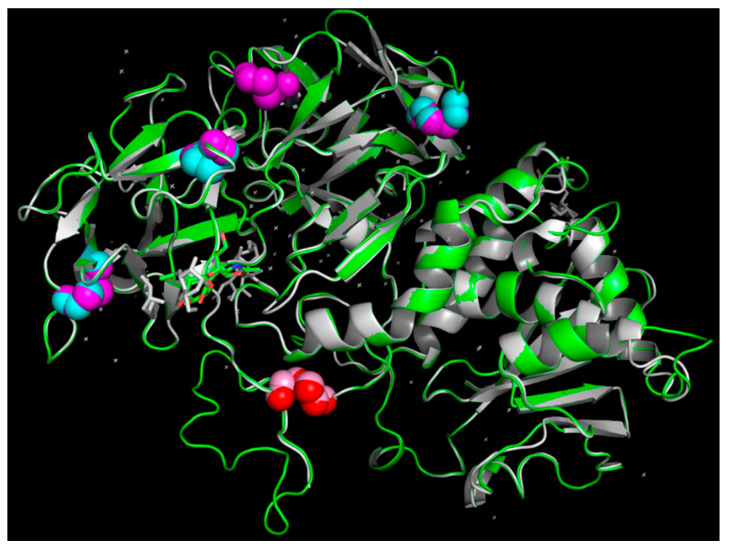
Comparison of the three-dimensional structure of malanin and cinnamonin III by PyMOL 2.3. The three-dimensional structures of malanin and cinnamonin III are shown in green and gray, respectively. The blue and magenta spheres are disulfide bonds of malanin and cinnamonin III within the B chain, and the disulfide bond in pink and red links together their A and B chains. The magenta spheres are disulfide bonds of cinnamonin III within the B chain.

The GMQE parameter was determined to indicate sufficient model quality. This parameter provides a quality estimation which combines properties from the target–template alignment and the template search method. The resulting GMQE score is expressed as a number between 0 and 1, reflecting the expected accuracy of a model built with that alignment and template and the coverage of the target. Higher numbers indicate higher reliability. The GMQE of cinnamonin III (2vlc.1.A) was found to have a score of 0.74, which is higher than those of ricin (0.68) and abrin (0.64). Therefore, the best three-dimensional structure of malanin was obtained using cinnamonin III (2vlc.1.A) as a template, as shown in Figure 3. And the comparison of the three-dimensional structures of malanin and cinnamonin III is shown in Figure 4.

As shown in Figure 4, the three-dimensional structures of malanin and cinnamonin III are shown in green and gray, respectively. The blue and magenta spheres are disulfide bonds of malanin and cinnamonin III within the B chain, and the disulfide bond in pink and red is linked to the A chain and B chain. The magenta spheres are disulfide bonds of cinnamonin III within the B chain.

**Figure 5:**
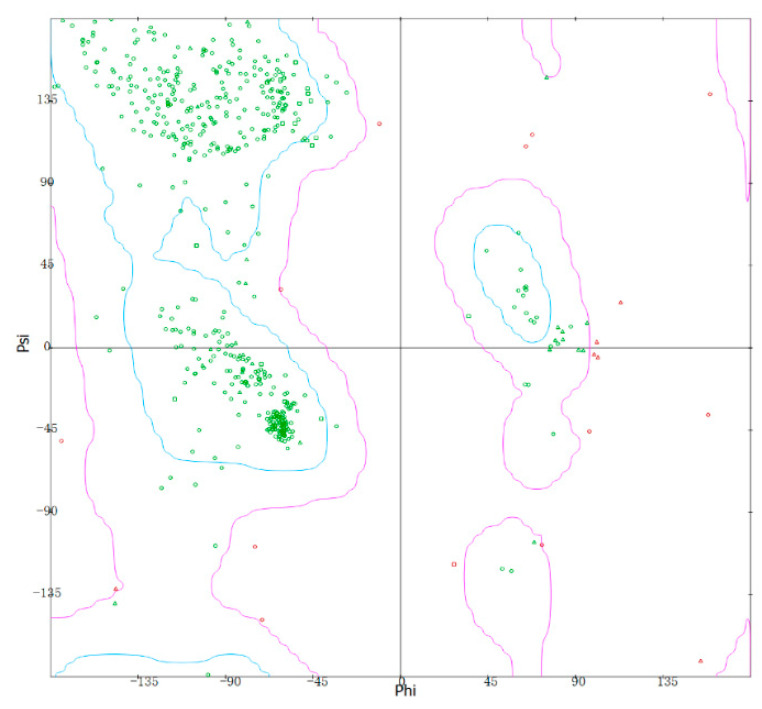
The Ramachandran plot of malanin by Discovery Studio 4.5. The most conformationally favorable region was within the blue line, and the feasible region was within the purple line (green dots). The area outside the purple line was not feasible (red dots).

The Ramachandran plot of malanin was produced by Discovery Studio 4.5 (Figure 5). The Ramachandran plot analysis of the likely structure showed that the blue region was the optimal region. The more amino acids in this region, the more reliable the structure; the purple region was the allowable region; and the dots in other regions (red dots) were amino acids with unreasonable psi–phi conformation and needed to be optimized. As shown in Figure 5, overall, 563 templates were found, of which 89.42% were in the most favorable regions (green dots), 7.24% being in additionally allowed regions. Only 3.34% of residues were in disallowed regions (red dots), indicating that the conformation of malanin was reliable.

### 2.4. Comparison between the Difference in Amino Acid Residues in Ricin and Malanin

The difference in amino acid residues in the three-dimensional structures between ricin and malanin was analyzed by PyMOL 2.3. As shown in Table 2, nonpolar, aliphatic R groups are abbreviated as NAL, and the static charge was 0; NAR means nonpolar, aromatic R groups, and the static charge was 0; PU means polar, uncharged R groups; “–” means negatively charged R groups; and “+” means positively charged R groups. There were 106 residues different in chain A in the three-dimensional structures between malanin and ricin, and 63 amino acid residues were different in chain B. In total, 9 of them were non-polar (0 ↔ 0), 146 of them were outside, and only 14 of them were inside.

Amino acid residues outside may have little effect on the active center. There are a total of 169 different amino acid residues in malanin and ricin, and only 14 different amino acid residues are inside; these are considered to potentially have an effect on the active center. This result shows that the spatial configurations of malanin and ricin are likely to be very similar.

### 2.5. Comparison between the Disulfide Bonds in Ricin and Malanin

**Figure 6:**
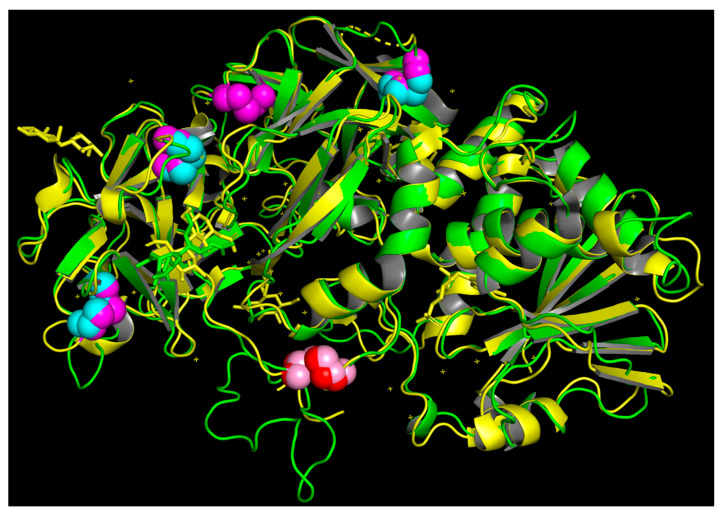
Comparison of the three-dimensional structures of malanin and ricin by PyMOL 2.3. The three-dimensional structures of malanin and ricin are shown in green and yellow, respectively. The blue and magenta spheres are disulfide bonds of malanin and ricin within the B chain, and the disulfide bond in pink and red links their A chain and B chain. The magenta spheres are disulfide bonds of ricin within the B chain.

It is known that mature ricin has five pairs of disulfide bonds, of which one pair links chain A to chain B. The cystine residues of mature ricin participate in the linkage from the B chain to the A chain, which was identified between cys259 in chain A and cys4 in chain B (red sphere in Figure 6, and four disulfide bridges were located between cys20 and cys39, cys62 and cys79, cys149 and cys163, and cys188 and cys205 in chain B (magenta spheres in Figure 6)) [22,61].

The alignment results of malanin and ricin in the primary structure showed that they both had 12 cysteine residues and were located in the same positions (the red rectangle in Figure 1). As shown in Figure 3, there were four disulfide bonds in the three-dimensional structure of malanin (three pairs in blue and one pair in pink), and the four disulfide bridges were located between cys20 and cys39, cys62 and cys79, and cys149 and cys163 in chain B of malanin (Figure 1).

The three-dimensional modeling overlays of malanin and ricin by PyMOL 2.3 are shown in Figure 6 in green and yellow, respectively. The blue and magenta spheres resemble disulfide bonds of malanin and ricin within the B chain, and the disulfide bonds in pink and red link the A and B chains together. The magenta spheres represent disulfide bonds of ricin within the B chain. As shown in Figure 6, there was high similarity between malanin and ricin, and the difference was that ricin had five disulfide bond pairs, whereas malanin had only four disulfide bond pairs. The positions of the four disulfide bond pairs were the same (three pairs in magenta and blue and one pair in red and pink), but cys188 and cys207 in the B chain of malanin did not form a disulfide bond, while ricin did (the sphere only in magenta). It is also shown in Figure 6 that malanin, like ricin, also consisted of two polypeptide chains that were crosslinked by one disulfide bond. The linkage of the B chain to the A chain was between cys258 in the A chain and cys4 in the B chain (the spheres in pink and red).

**Figure 7:**
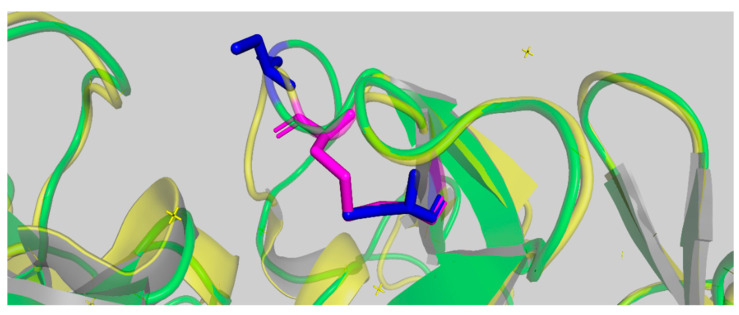
The bonding of cys188 and cys207 in malanin B chain with cys188 and cys205 in ricin B chain by PyMOL 2.3. The three-dimensional structures of malanin and ricin are shown in green and yellow, respectively. The two blue structures are the cys188 and cys207 within the malanin B chain, which did not form a disulfide bond. The magenta structure represents cys188 and cys205 within the ricin B chain, which formed a disulfide bond.

The bonding of cys188 and cys207 in the malanin B chain and of cys188 and cys205 in the ricin B chain were analyzed by PyMOL 2.3 (Figure 7). The three-dimensional structures of malanin and ricin are shown in green and yellow, respectively. The two blue regions are the cys188 and cys207 within the malanin B chain, which did not form a disulfide bond. The magenta color represents cys188 and cys205 within the ricin B chain, which formed a disulfide bond. From the results of the Ramachandran plot (Figure 5), only 3.34% of amino acid residues were in disallowed regions, and the amino acid residues were A3 P, A10 S, A14 S, A66 V, A91 Q, A132 D, A133 P, A222 D, A234 N, A235 Y, A261 P, A262 P, and A277 Y in the malanin A chain and A42 N, A84 V, A68 K, A230 H, and A255 N in the malanin B chain. The positions of the cys188 and cys207 in malanin B chain were in favorable regions. Therefore, whether these two cysteines can form disulfide bonds needs further experimental verification.

### 2.6. Active Site Analysis of Malanin

R180 and E177 of the ricin A chain provide the active site for ricin, and they have been shown to play a crucial role in the enzymatic inactivation of ribosomes [62].

**Figure 8:**
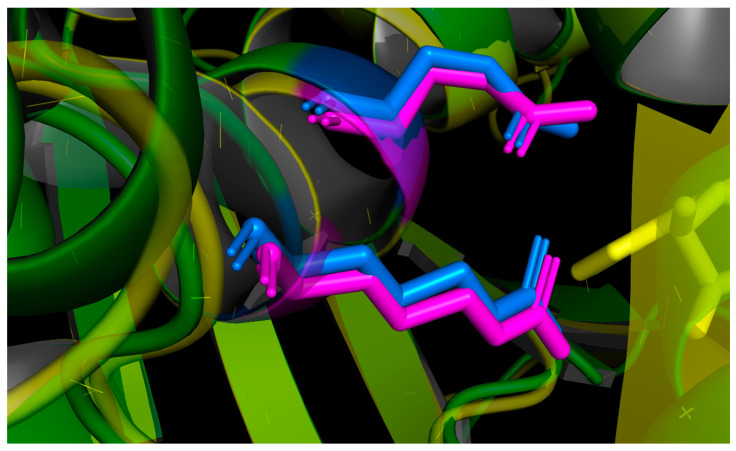
Active sites of malanin and ricin. The active sites of malanin and ricin are shown in blue and magenta, respectively. They are located in the same positions in their three-dimensional structures.

According to Figure 1, the active sites provided by R180, E177 in ricin and R176, E173 in malanin were located in the same positions in their primary structures (the active sites are framed by green rectangles in Figure 1). As shown in Figure 8, with the three-dimensional structures of malanin and ricin superimposed, the active sites R and E of malanin and ricin are shown in blue and magenta, respectively. They were located in the same positions in their three-dimensional structures.

## 3. Conclusions

Knowledge of the amino acid sequences and the 3D structural prediction of malanin and its similarity to ricin has high importance because of the potential use of malanin in medicine, such as for application in cancer treatment. Our previous research found that the LD_50_ of malanin by intraperitoneal injection in mice was 26.22 ± 9.03 µg/kg, while ricin was 2.4–36 µg/kg, indicating that the toxicity of malanin in mice is not much different from that of other type II RIPs such as ricin. If the key genes determining enzymatic activity and pulmonary vascular leakage in malanin could be mutated using site-directed mutagenesis technology, mutants of malanin with low toxicity, no vascular leakage, and good immunogenicity could be obtained. This could lay the foundation for using malanin as an immunotoxin to treat cancer, as well as provide a basis to develop and utilize natural plant resources in Yunnan, China.

## 4. Materials and Methods

### 4.1. Homologous Sequence Alignment of Malanin

The homology of amino acid sequences of malanin was searched in the non-redundant protein sequences (nr) database using the NCBI online Blastp (protein–protein BLAST) tool (https://blast.ncbi.nlm.nih.gov/Blast.cgi?PROGRAM=blastp&PAGE_TYPE=BlastSearch&LINK_LOC=blasthome (accessed on 21 November 2023)). The homologous alignment between malanin and ricin (UniProtKB/Swiss-Prot: P02879.1) was analyzed using DNAMAN 9.0 software [63,64].

### 4.2. The Phylogenetic Relationship of Malanin

The Clustalx (1.83) software was used to analyze the amino acid sequences of malanin and the homologous sequences of other species to construct a phylogenetic tree [65,66,67].

### 4.3. Homology Modeling of Malanin

Structure homology was computed by the SWISS-MODEL (Swiss Institute of Bioinformatics, Biozentrum, University of Basel, Basel, Switzerland) homology server [68,69], which relies on ProMod3, an in-house comparative modeling engine based on OpenStructure [70]. For validating the structure of malanin, Ramachandran plot (RC plot) analysis from Discovery Studio 4.5 was used [71,72,73].

### 4.4. Disulfide Bond Prediction

The cysteine residues in the primary structures of malanin and ricin were aligned using the DNAMAN 9.0 software. The positions of the disulfide bonds in the three-dimensional structure of malanin and ricin were analyzed by PyMOL 2.3 [74].

### 4.5. Active Sites of Malanin

The active sites in the primary and three-dimensional structure of malanin and ricin were analyzed by DNAMAN 9.0 and PyMOL 2.3, respectively [75,76].

## Figures and Tables

**Table 1:** Type II RIPs used to construct the phylogenetic tree shown in Figure 2.

Family	Species	Protein	Swiss-Prot/TrEMBL Accession Number	References
Olacaceae	*Malania oleifera*	Malanin	/	[12,13]
Olacaceae	*Ximenia americana* L.	Riproximin (Rpx)	Q2PA54	[28,29]
Euphorbiaceae	*Ricinus communis*	Ricin	P02879	[30]
Euphorbiaceae	*Ricinus communis*	Ricin Agglutinin (RCA)	P06750	[31]
Lauraceae	*Cinnamomum camphora*	Cinnamomin I	Q94BW5	[32]
Lauraceae	*Cinnamomum camphora*	Cinnamomin II	Q94BW4	[32]
Lauraceae	*Cinnamomum camphora*	Cinnamomin III	Q94BW3	[32]
Fabaceae	*Abrus precatorius*	Abrin-a	P11140	[33]
Fabaceae	*Abrus precatorius*	Abrin-b	Q06077	[34]
Fabaceae	*Abrus precatorius*	Abrin-c	P28590	[35]
Fabaceae	*Abrus precatorius*	Abrin-d	Q06076	[35]
Fabaceae	*Abrus precatorius*	APA	Q9M6E9	[31]
Iridaceae	*Iris hollandica*	IRAb	Q8W2E7	[36]
Iridaceae	*Iris hollandica*	IRAr	Q8W2E8	[36]
Sambucaceae	*Sambucus ebulus*	Ebulin l	Q9AVR2	[37]
Sambucaceae	*Sambucus nigra*	Nigrin b	P33183	[38]
Sambucaceae	*Sambucus nigra*	Nigrin 1	Q8GT32	[39]
Sambucaceae	*Sambucus nigra*	Nigrin f	O04367	[39]
Sambucaceae	*Sambucus nigra*	SNA-IV	O04366	[40]
Sambucaceae	*Sambucus nigra*	SNA-IVl	Q945S4	[41]
Sambucaceae	*Sambucus nigra*	SNA-V1	Q945S2	[42]
Sambucaceae	*Sambucus nigra*	SNAlm	Q8GTA5	[43]
Sambucaceae	*Sambucus nigra*	SNAld	Q8GTA6	[44]
Sambucaceae	*Sambucus nigra*	SNAIf	O22415	[45]
Sambucaceae	*Sambucus nigra*	SNAI	Q41358	[46]
Sambucaceae	*Sambucus nigra*	SNAI’	P93543	[47]
Sambucaceae	*Sambucus nigra*	SNLRP1	O04072	[48]
Sambucaceae	*Sambucus nigra*	SNLRP2	O04071	[48]
Sambucaceae	*Sambucus sieboldiana*	Sieboldin a (SSA)	D25317 (GenBank)	[49]
Liliaceae	*Polygonatum multiflorum*	RIPt	Q9M653	[50]
Liliaceae	*Polygonatum multiflorum*	RIPm	Q9M654	[50]
Viscaceae	*Viscum album coloratum*	VCA	Q8W243	[51]
Viscaceae	*Viscum album*	Mistletoe lectin (ML) I	P81446	[52]
Viscaceae	*Viscum album*	ML II	Q6H266	[53]
Viscaceae	*Viscum album*	ML III	P82683	[54]
Viscaceae	*Viscum album*	ML IV	Q6ITZ3	[55]
Cucurbitaceae	*Momordica charantia* L.	MCL1	B7X8M2	[56]
Cucurbitaceae	*Trichosanthes anguina* L.	SGSL	U3KRF8	[57]
Passifloraceae	*Adenia volkensii* Harms	Volkensin	Q70US9	[58]

**Table 2:** The difference in amino acid (AA) residues in the three-dimensional structures between ricin and malanin by PyMOL 2.3.

Amino Acid Residues in Chain A											
AA Position	Malanin	Ricin	Changes	Charge	Spatial Location in Malanin	AA Position	Malanin	Ricin	Changes	Charge	Spatial Location in Malanin
4	K	I	+ ↔ NAL	+ ↔ 0	outside	145	D	S	− ↔ PU	− ↔ PU	outside
10	S	A	PU ↔ NAL	PU ↔ 0	outside	149	H	Y	+ ↔ NAR	+ ↔ 0	outside
12	T	A	PU ↔ NAL	PU ↔ 0	outside	150	R	Y	+ ↔ NAR	+ ↔ 0	outside
13	V	T	NAL↔ PU	0↔ PU	outside	152	G	T	NAL ↔ PU	0 ↔ PU	outside
14	S	V	PU ↔ NAL	PU ↔ 0	outside	153	T	G	PU ↔ NAL	PU ↔ 0	outside
15	K	Q	+ ↔ PU	+ ↔ PU	outside	154	H	G	+ ↔ NAL	+ ↔ 0	outside
19	R	T	+ ↔ PU	+ ↔ PU	outside	155	G	T	NAL↔ PU	0 ↔ PU	outside
23	Q	R	PU ↔ +	PU ↔ +	outside	156	D	Q	− ↔ PU	− ↔ PU	outside
24	S	A	PU ↔ NAL	PU ↔ 0	outside	157	R	L	+ ↔ NAL	+ ↔ 0	outside
27	D	G	− ↔ NAL	− ↔ 0	outside	158	A	P	NAL↔ PU	0 ↔ PU	outside
30	A	T	NAL ↔ PU	0 ↔ PU	outside	159	K	T	+ ↔ PU	+ ↔ PU	outside
32	P	G	PU ↔ NAL	PU ↔ 0	outside	164	L	F	NAL↔ NAR	0 ↔ 0	/
33	Q	A	PU ↔ NAL	PU ↔ 0	outside	169	L	Q	NAL↔PU	0 ↔ PU	inside
35	R	V	+ ↔ NAL	+ ↔ 0	outside	178	R	Q	+ ↔ PU	+ ↔ PU	inside
38	G	E	NAL ↔ −	0 ↔ −	outside	182	N	G	PU ↔ NAL	PU ↔ 0	inside
43	F	P	NAR ↔ PU	0 ↔ PU	outside	185	A	R	NAL↔ +	0 ↔ +	outside
44	D	N	− ↔ PU	− ↔ PU	outside	186	R	T	+ ↔ PU	+ ↔ PU	outside
45	P	R	PU ↔ +	PU ↔ +	outside	187	T	R	PU ↔ +	PU ↔ +	outside
46	N	V	PU ↔ NAL	PU ↔ 0	outside	189	S	R	PU ↔ +	PU ↔ +	outside
47	N	G	PU ↔ NAL	PU ↔ 0	outside	190	S	Y	PU ↔ NAR	PU ↔ 0	outside
50	D	I	− ↔ NAL	− ↔ 0	outside	191	H	N	+ ↔ PU	+ ↔ PU	outside
62	N	H	PU ↔ +	PU ↔ +	outside	192	G	R	NAL ↔ +	0 ↔ +	outside
65	D	L	− ↔ NAL	− ↔ 0	outside	193	Y	R	NAR ↔ +	0 ↔ +	outside
66	V	S	NAL ↔ PU	0 ↔ PU	outside	194	G	S	NAL↔ PU	0 ↔ PU	outside
84	R	G	+ ↔ NAL	+ ↔ 0	outside	195	T	A	PU ↔ NAL	PU ↔ 0	outside
85	G	N	NAL↔ PU	0 ↔ PU	outside	199	N	D	PU ↔ −	PU ↔ −	outside
86	E	S	− ↔ PU	− ↔ PU	outside	200	G	P	NAL ↔ PU	0 ↔ PU	outside
87	S	A	PU ↔ NAL	PU ↔ 0	outside	201	A	S	NA L↔ PU	0 ↔ PU	outside
91	Q	H	PU ↔ +	PU ↔ +	outside	204	R	T	+ ↔ PU	+ ↔ PU	outside
92	D	P	− ↔ PU	− ↔ PU	outside	206	V	E	NAL ↔ −	0 ↔ −	outside
93	A	D	NAL↔ −	0 ↔ −	outside	207	R	N	+ ↔ PU	+ ↔ PU	outside
95	H	Q	+ ↔ PU	+ ↔ PU	outside	208	R	S	+ ↔ PU	+ ↔ PU	outside
96	V	E	NAL↔ −	0 ↔ −	outside	210	D	G	− ↔ NAL	− ↔ 0	outside
98	F	I	NAR↔ NAL	0 ↔ 0	/	218	H	E	+ ↔ −	+ ↔ −	outside
100	N	H	PU ↔ +	PU ↔ +	outside	220	E	N	− ↔ PU	− ↔ PU	outside
105	T	V	PU ↔ NAL	PU ↔ 0	outside	221	E	Q	− ↔ PU	− ↔ PU	outside
107	Q	R	PU ↔ +	PU ↔ +	outside	222	D	G	− ↔ NAL	− ↔ 0	outside
110	L	F	NAL↔ NAR	0 ↔ 0	/	224	S	F	PU ↔ NAR	PU ↔ 0	outside
111	T	A	PU ↔ NAL	PU ↔ 0	outside	225	F	A	NAR ↔ NAL	0 ↔ 0	/
117	A	D	NAL↔ −	0 ↔ −	outside	233	S	R	PU ↔ +	PU ↔ +	outside
118	D	R	− ↔ +	− ↔ +	outside	235	Y	G	NAR ↔ NAL	0 ↔ 0	/
120	L	E	NAL↔ −	0 ↔ −	outside	236	V	S	NAL ↔ PU	0 ↔ PU	outside
121	G	Q	NAL↔ PU	0 ↔ PU	outside	237	P	K	PU ↔ +	PU ↔ +	outside
125	L	N	NAL↔ PU	0 ↔ PU	outside	241	S	Y	PU ↔ NAR	PU ↔ 0	inside
126	S	L	PU ↔ NAL	PU ↔ 0	outside	242	N	D	PU ↔ −	PU ↔ −	inside
127	D	R	− ↔ +	− ↔ +	outside	244	M	S	NAL ↔ PU	0 ↔ PU	inside
128	L	E	NAL↔ −	0 ↔ −	outside	245	P	I	PU ↔ NAL	PU ↔ 0	inside
129	D	N	− ↔ PU	− ↔ PU	outside	246	E	L	− ↔ NAL	− ↔ 0	inside
130	R	I	+ ↔ NAL	+ ↔ 0	outside	249	A	P	NAL↔ PU	0 ↔ PU	inside
131	L	E	NAL↔ −	0 ↔ −	outside	250	T	I	PU ↔ NAL	PU ↔ 0	inside
137	I	N	NAL↔ PU	0 ↔ PU	outside	257	I	R	NAL ↔ +	0 ↔ +	outside
138	Q	G	PU ↔ NAL	PU ↔ 0	outside	259	E	A	− ↔ NAL	− ↔ 0	outside
142	S	E	PU ↔ −	PU ↔ −	outside	260	K	P	+ ↔ PU	+ ↔ PU	outside
**Amino Acid Residues in Chain B**											
**AA Position**	**Malanin**	**Ricin**	**Changes**	**Charge**	**Spatial Location** **in Malanin**	**AA Position**	**Malanin**	**Ricin**	**Changes**	**Charge**	**Spatial Location** **in Malanin**
3	T	V	PU ↔ NAL	PU ↔ 0	outside	157	N	G	PU ↔ NAL	PU ↔ 0	outside
5	T	M	PU ↔ NAL	PU ↔ 0	outside	158	D	Q	− ↔ PU	− ↔ PU	outside
7	E	P	− ↔ PU	− ↔ PU	outside	165	V	S	NAL↔ PU	0 ↔ PU	outside
9	F	P	NAR ↔ PU	0 ↔ PU	outside	166	D	S	− ↔ PU	− ↔ PU	outside
10	T	I	PU ↔ NAL	PU ↔ 0	outside	177	P	A	PU ↔ NAL	PU ↔ 0	inside
25	G	D	NAL↔ −	0 ↔ −	outside	179	R	G	+ ↔ NAL	+ ↔ 0	inside
27	F	R	NAR↔ +	0 ↔ +	outside	185	E	Q	− ↔ PU	− ↔ PU	outside
29	N	H	PU ↔ +	PU ↔ +	outside	189	L	N	NAL↔ PU	0 ↔ PU	outside
32	D	N	− ↔ PU	− ↔ PU	outside	193	Y	S	NAR ↔ PU	0 ↔ PU	outside
33	P	A	PU ↔ NAL	PU ↔ 0	outside	194	Y	D	NAR↔ −	0 ↔ −	outside
35	I	Q	NAL ↔ PU	0 ↔ PU	outside	195	E	S	− ↔ PU	− ↔ PU	outside
43	A	T	NAL ↔ PU	0 ↔ PU	outside	197	Q	I	PU ↔ NAL	PU ↔ 0	outside
55	G	N	NAL↔ PU	0↔ PU	outside	198	S	R	PU ↔ +	PU ↔ +	outside
60	K	N	+ ↔ PU	+ ↔ PU	outside	200	D	T	− ↔ PU	− ↔ PU	outside
73	S	V	PU ↔ NAL	PU ↔ 0	outside	202	T	V	PU ↔ NAL	PU ↔ 0	outside
81	A	N	NAL ↔ PU	0 ↔ PU	outside	203	I	K	NAL ↔ +	0 ↔ +	outside
91	E	Q	− ↔ PU	− ↔ PU	outside	205	N	L	PU ↔ NAL	PU ↔ 0	outside
108	S	A	PU ↔ NAL	PU ↔ 0	outside	206	I	S	NAL ↔ PU	0 ↔ PU	outside
110	E	T	− ↔ PU	− ↔ PU	outside	207	A	C	NAL ↔ PU	0 ↔ PU	outside
115	D	G	− ↔ NAL	− ↔ 0	outside	208	S	G	PU ↔ NAL	PU ↔ 0	outside
122	V	T	NAL↔ PU	0↔ PU	outside	210	S	A	PU ↔ NAL	PU ↔ 0	outside
124	N	I	PU ↔ NAL	PU ↔ 0	outside	215	R	G	+ ↔ NAL	+ ↔ 0	outside
126	S	A	PU ↔ NAL	PU ↔ 0	outside	216	E	Q	− ↔ PU	− ↔ PU	outside
127	S	V	PU ↔ NAL	PU ↔ 0	outside	221	Q	K	PU ↔ +	PU ↔ +	inside
128	R	S	+ ↔ PU	+ ↔ PU	outside	230	H	Y	+ ↔ NAR	+ ↔ 0	outside
133	A	P	NAL ↔ PU	0 ↔ PU	inside	231	L	S	NAL↔ PU	0 ↔ PU	outside
136	E	N	− ↔ PU	− ↔ PU	outside	239	R	A	+ ↔NAL	+ ↔ 0	outside
145	W	V	NAR↔ NAL	0↔ 0	/	252	F	L	NAR ↔ NAL	0 ↔ 0	/
147	F	L	NAR ↔ NAL	0 ↔ 0	/	255	N	D	PU ↔ −	PU ↔ −	outside
148	R	Y	+ ↔ NAR	+ ↔ 0	outside	259	Q	I	PU ↔ NAL	PU ↔ 0	outside
149	D	G	− ↔ NAL	− ↔ 0	outside	261	F	L	NAR ↔ NAL	0 ↔ 0	/
156	G	S	NAL ↔ PU	0 ↔ PU	outside						

Note: NAL = nonpolar, aliphatic R groups, 0; NAR = nonpolar, aromatic R groups, 0; PU = polar, uncharged R groups; − = negatively charged R groups; + = positively charged R groups.

## Data Availability

All data can be supplied on request.
